# Association of calcium sensing receptor polymorphisms at rs1801725 with circulating calcium in breast cancer patients

**DOI:** 10.1186/s12885-017-3502-3

**Published:** 2017-08-02

**Authors:** Li Wang, Sarrah E. Widatalla, Diva S. Whalen, Josiah Ochieng, Amos M. Sakwe

**Affiliations:** 1 Department of Biochemistry and Cancer Biology, School of Graduate Studies and Research, Meharry Medical College, Nashville, TN 37208 USA; 2 Vanderbilt Center for Quantitative Sciences, Department of Biostatistics, Vanderbilt University, Nashville, TN USA

**Keywords:** Calcium-sensing receptor, Single nucleotide polymorphism, Cancer-induced hypercalcemia, Breast cancer, Genome-wide association studies

## Abstract

**Background:**

Breast cancer (BC) patients with late-stage and/or rapidly growing tumors are prone to develop high serum calcium levels which have been shown to be associated with larger and aggressive breast tumors in post and premenopausal women respectively. Given the pivotal role of the calcium sensing receptor (CaSR) in calcium homeostasis, we evaluated whether polymorphisms of the CASR gene at rs1801725 and rs1801726 SNPs in exon 7, are associated with circulating calcium levels in African American and Caucasian control subjects and BC cases.

**Methods:**

In this retrospective case-control study, we assessed the mean circulating calcium levels, the distribution of two inactivating CaSR SNPs at rs1801725 and rs1801726 in 199 cases and 384 age-matched controls, and used multivariable regression analysis to determine whether these SNPs are associated with circulating calcium in control subjects and BC cases.

**Results:**

We found that the mean circulating calcium levels in African American subjects were higher than those in Caucasian subjects (*p* < 0.001). As expected, the mean calcium levels were higher in BC cases compared to control subjects (*p* < 0.001), but the calcium levels in BC patients were independent of race. We also show that in BC cases and control subjects, the major alleles at rs1801725 (G/T, A986S) and at rs1801726 (C/G, Q1011E) were common among Caucasians and African Americans respectively. Compared to the wild type alleles, polymorphisms at the rs1801725 SNP were associated with higher calcium levels (*p* = 0.006) while those at rs1801726 were not. Using multivariable linear mixed-effects models and adjusting for age and race, we show that circulating calcium levels in BC cases were associated with tumor grade (*p* = 0.009), clinical stage (*p* = 0.003) and more importantly, with inactivating mutations of the CASR at the rs1801725 SNP (*p* = 0.038).

**Conclusions:**

These data suggest that decreased sensitivity of the CaSR to calcium due to inactivating polymorphisms at rs1801725, may predispose up to 20% of BC cases to high circulating calcium-associated larger and/or aggressive breast tumors.

## Background

Breast cancer (BC) is frequently diagnosed as an aggressive disease with poor prognosis especially in younger and women of African ancestry. The underlying mechanisms and factors that promote the aggressive behavior of BC in this subset of patients remain poorly understood. Among the potential factors is the development of cancer-induced hypercalcemia (CIH), an often overlooked metabolic disorder which is inevitable in late-stage, metastatic and aggressive BC [1, 2]. Available evidence reveals that serum calcium levels are elevated in women with untreated BC [3], and that high serum calcium levels are associated with aggressive breast tumors among premenopausal and/or overweight women [4], and larger breast tumors among postmenopausal women [5]. However, whether these high calcium associated breast cancer outcomes are related to the functional status of the calcium sensing receptor (CaSR) [6] remains unclear.

As a major component of the calcium homoeostatic system [7], the CaSR contributes to the development of CIH by promoting the growth and metastatic properties of tumor cells [8, 9] and/or by promoting the secretion of tumor cell-derived osteolytic factors such as parathyroid hormone-related protein (PTHrP) [10–12]. However,, in bone and mineral ion disorders, the CaSR is invariably mutated into several loss- or gain-of-function variants [13, 14] and these are respectively associated with hypercalcemia and hypocalcemia [15, 16]. The CaSR proteins with loss-of-function or inactivating mutations in the coding sequence have been shown to be less sensitive to calcium [17, 18] and linked with familial hypocalciuric hypercalcemia, more severe primary hyperparathyroidism, and the risk of kidney stones [13, 15, 19–21].

Among the several mutations in the cytosolic domain of the CASR, single nucleotide polymorphisms (SNP) at rs1801725 and rs1801726 in exon 7 are loss-of-function or inactivating mutations. Polymorphisms at these SNPs have not only been shown to lead to reduced sensitivity (right-shifted response) to calcium [22] but are also important in the development of hypercalcemia in a mouse model of squamous cell lung carcinoma [16]. Although the CaSR is pivotal in calcium homeostasis, its contribution in the previously reported association of high calcium with larger or more aggressive breast tumors remain unclear. In this study, we investigated whether these CASR SNPs are associated with higher circulating calcium levels in control versus BC Caucasian and African American women. Our data reveal that CASR polymorphisms at rs1801725 but not at rs1801726 SNP are associated with calcium and suggest that polymorphisms at rs1801725 in about 20% of BC cases, underlie, at least in part, the previously reported association of high circulating calcium with BC progression into larger and/or aggressive tumors.

## Methods

### Ethical considerations and study subjects

This study was classified by the Meharry Medical College and Vanderbilt University institutional review boards as non-human subject research and required a satisfactorily completed Data Use Agreement for the Vanderbilt University DNA biorepository (BioVU) and de-identified patient records (Synthetic Derivative) databases. BC cases were identified from these databases using the following search criteria: ICD-9 code 174 (neoplasms of the female breast), tumor registries, calcium assay data, gender (= female), race (= Caucasian or African American) and genome-wide association studies (GWAS) genotyping data. For GWAS we focused on polymorphisms at codons 986 (rs1801725) and 1011 (rs1801726) in exon 7 (cytosolic domain) of the CASR as these correspond to inactivating mutant CaSRs with decreased sensitivity to calcium. De-identified information about the disease grade and/or stage was obtained from tumor registries while calcium assay data were extracted from the Synthetic Derivative database. For age-matched control records, only records with calcium and GWAS data with no evidence of any form of malignancy were retained for the study.

### Statistical analysis

Descriptive statistics are presented as the median with interquartile range (IQR) and mean +/− SD for calcium assay data; and frequencies (percentages) for genotypes and allele frequencies. The distribution of CASR genotypes and alleles frequency in the groups (control versus BC cases or Caucasian versus African American) was compared using Pearson Chi-squared test. The primary outcome was circulating or serum calcium levels. The average calcium levels as well as the genotypes at the two SNPs between controls and BC cases or African Americans (Blacks) and Caucasians (Whites) were compared using Wilcoxon rank sum test. The interaction between calcium levels and genotypes at the two SNPs was analyzed using the linear mixed-effects model (additive and co-dominant) fit by restricted maximum likelihood (REML) and adjusting for BC stage, grade, race, and age at diagnosis. The Fisher’s exact test was used to test the relationship between polymorphisms at the two SNPs and BC stage and grade. All analyses were performed using the statistical software R version 3.1.2 (https://wwwr-project.org/) and a *p* < 0.05 was considered to be statistically significant. To estimate the power of our analysis especially for the continuous variable calcium, we assumed that the standard deviation was 0.5 and a Type I error probability of 5%. Using these parameters, we required 199 cases and 199 controls to detect a difference of 0.163 in calcium levels between two groups with a 90% power.

## Results

### BioVU search strategy, inclusion criteria and data extraction

The BioVU and Synthetic Derivative databases at Vanderbilt University have been successfully used to characterize gene-disease associations in multiple diseases [23], to identify predictors of diseases [24, 25] and to predict the risk of disease [26, 27]. Based on an initial search for records with calcium assay data, these databases contained 2111 records from African Americans and 2996 records from Caucasians. Our search criteria led to the identification of 359 BC cases with calcium assay data of which 199 were linked to genotyping data. This represented 58 records (29%) from African American and 141 records (71%) from Caucasian BC patients with a mean age of 54.9 ± 4.4 years. The BC cases comprised BC patients with varying degrees of disease severity. As expected most of the cases were patients with grades 2 and 3 or clinical stages I and II. Applying our exclusion criteria to search these databases, we identified 384 records as age and genetic ancestry-matched controls with calcium assay and genotyping data. This included 113 (29%) and 271 (71%) records from African American and Caucasian subjects respectively, with a mean age of 56.1 ± 3.2 years.

### Frequency of CaSR alleles in breast cancer cases

Analysis of the frequency of CASR alleles in the entire dataset (Table 1) revealed that the majority of these women (*n* = 583) expressed the wild type CASR at the rs1801725 SNP (79%) and at the rs1801726 SNP (87%). Table 1 also shows that the distribution of the major alleles at these loci was similar in the control subjects and in BC cases. As such, the frequency of the A986S (G/T) variant at the rs1801725 SNP was 19% in control versus 21% in BC cases, while the frequency of the Q1011E (C/G) variant at the rs1801726 SNP was 13% versus 10% in the control subjects and BC cases respectively. Overall, the A986S (G/T) variant was more common (20%) than the Q1011E (C/G) variant of the receptor (12%). Stratification of the distribution of the CASR variants by race revealed that the A986S CASR variant was common among Caucasians compared to African Americans (24% versus 9%) while the Q1011E CASR variant was common among African American subjects compared to Caucasians (24% versus 7%). All other alleles at the two SNPs were infrequent among both control or BC cases and the two racial groups.

**Table 1:** Distribution of rs1801725 and rs1801726 CaSR alleles in control subjects versus breast cancer cases

SNP ID	Genotype	Mutant CaSR	Controls *n* = 384		Breast cancer cases *n* = 199		All samples *n* = 583	
			n	%	n	%	n	%
RS1801725	G/G	A986A	304	79%	154	77%	458	79%
	G/T	A986S	74	19%	41	21%	115	20%
	T/T	S986S	6	2%	4	2%	10	2%
RS1801726	C/C	Q1011Q	328	85%	178	89%	506	87%
	C/G	Q1011E	50	13%	19	10%	69	12%
	G/G	E1011E	6	2%	2	1%	8	1%
			Blacks *N* = 171		Whites *N* = 412		All samples *N* = 583	
RS1801725	G/G	A986A	154	90%	304	74%	458	79%
	G/T	A986S	16	9%	99	24%	115	20%
	T/T	S986S	1	1%	9	2%	10	2%
RS1801726	C/C	Q1011Q	121	71%	385	93%	506	87%
	C/G	Q1011E	42	24%	27	7%	69	12%
	G/G	E1011E	8	5%	0	0%	8	1%

### Circulating calcium levels in control versus breast cancer cases

**Fig. 1:**
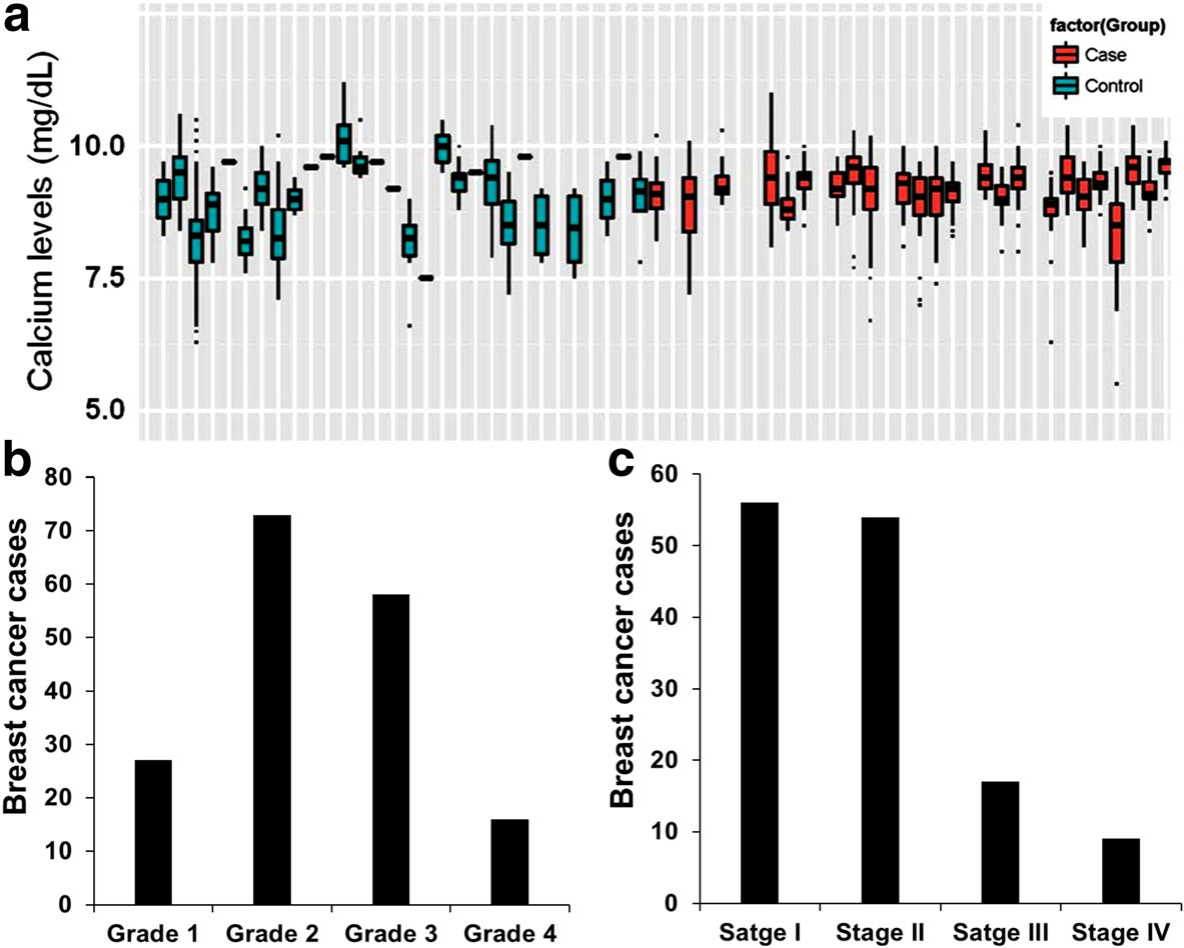
Serum calcium values and distribution of breast cancer cases by disease severity**. a** Representative box plots of the multiple serum calcium values from control and breast cancer cases. Each box plot represents the median and the Range (lower or 25th percentile and upper or 75th percentile) of the multiple circulating calcium concentrations from a single control subject (green) or a single breast cancer case (*red*). **b** and **c** Distribution of breast cancer cases according to tumor grades (**b**) and clinical stage (**c**)

For each study subject or identified record, multiple calcium measurements were obtained from distinct clinic visits. The recorded calcium levels varied within a narrow range for each control subject or BC case with some outliers (Fig. 1a). The BC cases comprised BC patients with varying degrees of disease severity. As expected most of the cases were patients with grades 2 and 3 (Fig. 1b) or clinical stages I and II Fig. 1c). It should be noted that diagnosis of most of the BC patients was indicated long before the establishment of BioVU and Synthetic Derivative databases. For these reasons, the mean serum calcium levels or the median and the 25th and 75th percentiles for each subject were used for our analysis. As depicted in Table 2 and as expected, the mean circulating calcium level in BC cases was significantly higher than that in control subjects (*P* < 0.001). Table 2 also shows that among the control subjects, the mean circulating calcium levels were significantly higher in African American subjects than in Caucasian subjects (*n* = 384; *p* < 0.001). However, among the BC cases, the mean circulating calcium levels were not significantly different between Caucasian and African American cases (*n* = 199; *p* = 0.51). This suggests that cancer-induced hypercalcemia is not associated with race.

**Table 2:** Circulating calcium levels in control subjects and breast cancer cases expressing inactivating CaSR mutants

Disease Status	Control subjects			Breast cancer cases			
	n	Median Calcium^a^	Range^b^	n	Median calcium	Range	*p*-Value^c^
	384	9.09 ± 0.54	8.76–9.45	199	9.29 ± 0.40	9.02–9.52	<0.001
Race	African Americans			Caucasians			
All Samples	171	9.28 ± 0.45	9.02–9.54	412	9.11 ± 0.55	8.80–9.46	<0.001
CONTROL	113	9.26 ± 0.47	9.00–9.53	271	9.02 ± 0.55	8.67–9.37	<0.001
BC Cases	58	9.30 ± 0.39	9.03–9.60	141	9.29 ± 0.40	9.02–9.50	0.51

^a^mean circulating calcium ±1SD from multiple measurements over variable time periods. ^b^the lower and upper quartiles of circulating calcium levels for each group. ^c^Significance of the difference in circulating calcium between controls and breast cancer cases or between African American and Caucasian control subjects or BC cases. The *p*-values were calculated using the Wilcoxon rank sum test from the median with interquartile ranges for each group

### Inactivating CaSR mutants and circulating calcium levels in breast cancer cases

To determine whether the two inactivating CaSR SNPs are associated with calcium, we first compared the mean circulating calcium levels in control subjects and BC cases, stratified according to the CASR genotypes at the two SNPs. As shown in Table 3, the mean circulating calcium levels were significantly higher in all subjects expressing the G/T (*n* = 115; *p* = 0.006) and T/T (*n* = 10; *p* = 0.024) variants of the CASR at the rs1801725 SNP compared to subjects expressing the wild type receptor. Surprisingly, variants of the receptor at the rs1801726 SNP were not associated with higher serum calcium levels.

**Table 3:** Circulating calcium levels in control subjects and breast cancer cases expressing inactivating CaSR mutants

SNP ID	Genotype	n	Mean^a^	Range^b^	*p*-value^c^
RS1801725	G/G (AA)	458	9.13 ± 0.51	8.83–9.46	
	G/T (AS)	115	9.25 ± 0.48	9.00–9.56	0.006
	T/T (SS)	10	9.48 ± 0.50	9.29–9.75	0.024
RS1801726	C/C (QQ)	506	9.15 ± 0.52	8.86–9.48	
	C/G (QE)	69	9.20 ± 0.41	8.92–9.47	0.51
	G/G (EE)	8	9.10 ± 0.69	9.07–9.50	0.75

^a^Mean circulating calcium (mg/dL) from multiple measurements over variable time periods. ^b^The lowest and highest mean circulating calcium levels for each genotype. ^c^Significance in the difference in circulating calcium in subjects expressing the wild type receptor to those expressing polymorphic variants at the two SNPs. The *p*-values were calculated using the Wilcoxon rank sum test from the median with interquartile ranges for each genotype

We next determined whether the genotypes of the CASR at the two SNPs influenced circulating calcium levels differently in Caucasian and African American women expressing the major alleles at the two SNPs. Table 4 shows that even though the mean circulating calcium levels were higher in African American than in Caucasian control subjects, the higher calcium levels in African American women were not associated with the expression of mutant CaSRs at the two SNPs. On the other hand, serum calcium levels in control (*p* = 0.002) and BC (*p* = 0.034) Caucasian women expressing the G/T allele at the rs1801725 SNP were significantly higher than those in Caucasian subjects expressing the wild type receptor.

**Table 4:** Circulating calcium levels in control subjects and breast cancer cases expressing inactivating CaSR mutants stratified by race

Control subjects						Breast cancer cases		
SNP	Race	Genotype	n	Calcium (mg/dl)	*p*-value^a^	n	Calcium (mg/dl)	*p*-value^b^
rs1801725	Whites	G/G	203	8.96 ± 0.55		101	9.24 ± 0.39	
		G/T	63	9.16 ± 0.51	0.002	36	9.40 ± 0.43	0.034
	Blacks	G/G	101	9.26 ± 0.48		53	9.30 ± 0.39	
		G/T	11	9.25 ± 0.39	0.83	5	9.26 ± 0.42	0.82
rs1801726	Whites	C/C	253	9.02 ± 0.56		132	9.29 ± 0.41	
		C/G	18	9.06 ± 0.51	0.69	9	9.29 ± 0.36	0.95
	Blacks	C/C	75	9.31 ± 0.48		46	9.29 ± 0.40	
		C/G	32	9.21 ± 0.36	0.21	10	9.33 ± 0.39	0.95

Difference in the mean circulating calcium between subjects expressing the wild type and the mutant receptor in control (^a^) or breast cancer cases (^b^). The *p*-values were calculated using the Wilcoxon rank sum test from the median with interquartile ranges for each group

### Association of calcium levels with CaSR variants and breast cancer outcomes

The interaction between the two SNPs and circulating calcium levels in BC cases was further evaluated using the multivariable co-dominant and additive linear mixed-effects models. Table 5 shows that after adjusting for age and race, and as expected, circulating calcium levels in BC cases were associated with tumor grade (*p* = 0.009) and clinical stage (*p* = 0.003). More importantly, and consistent with data in Table 4, inactivating mutations of the CASR at the rs1801725 SNP were significantly associated with circulating calcium (*p* = 0.038) while inactivating mutations at the rs1801726 SNP were not associated with circulating calcium (*p* = 0.942). Together with data in Table 1, these data suggest that polymorphic CASR variants at the rs1801725 SNP contribute to the development of breast cancer-induced hypercalcemia and consequently, the high circulating calcium associated progression of BC into larger or aggressive breast tumors [3–5] in the up to 20% of women with mutations at the rs1801725 SNP.

**Table 5:** Interaction between race, tumor grade, clinical stage and CaSR SNPs with circulating calcium levels in breast cancer patients

Parameter	Additive model		Co-dominant model	
	F-value	*P*-value	F-value	*P*-value
AGE AT DIAGNOSIS	0.27	0.6045	0.28	0.5981
RACE	0.40	0.5281	0.39	0.5318
BC GRADE	3.40	0.0088	3.38	0.0091^a^
TNM CLINICAL STAGE	4.02	0.0029	3.99	0.0031^a^
RS1801725	6.70	0.0104	3.33	0.0379^a^
RS1801726	0.80	0.7716	0.06	0.9420

^a^Statistically significant association between parameter and circulating calcium

## Discussion

Cancer-induced hypercalcemia (CIH) is a metabolic syndrome which inevitably develops in patients with late-stage BC and/or metastasis to skeletal tissues [11, 28, 29]. On the other hand, in most patients with low grade tumors, CIH is either undetected or diagnosed as mild, non-life threatening increase in circulating calcium. Nevertheless, such mild increases in circulating calcium levels may substantially promote disease progression by activating the CaSR and/or other calcium dependent oncogenic pathways. Our findings that only polymorphisms in the rs1801725 SNP of the receptor are associated with higher calcium levels suggest that mutations in codon 986 in exon 7 of the CASR are associated with BC outcomes driven by higher than normal circulating calcium levels such as larger and more aggressive breast tumors.

High calcium mediated activation of the CaSR not only leads to increased proliferation and migration of BC cells [8] but also increased secretion of tumor cell-derived PTHrP [8, 9] which contributes to the vicious osteolytic cycle [28, 30]). Alteration of the function of the CaSR by pharmacological inhibition of its activity e.g. using calcilytic agents has been shown to inhibit cancer cell proliferation and metastasis [31]. Although decreased sensitivity of the receptor may be associated with reduced activity at physiologically normal calcium levels, inactivating mutant CaSRs require higher circulating calcium levels to effectively activate downstream effectors. It is possible that a combination of inactivating mutant CaSR expression and progressive increase in circulating cancer cell-derived osteolytic factors contribute to the observed higher circulating calcium in BC cases. Analysis of the distribution of the common CaSR alleles at rs1801725 and rs1801726 SNPs among BC cases confirmed previous reports that the A986S CaSR variant is common among Caucasians while the Q1011E variant is common among African Americans [17, 32–36]. Therefore, in both the control and BC cohorts, polymorphic variants in exon 7 of the CaSR occur with distinct frequencies among African Americans and Caucasians but the implication, if any, of the CaSR variants in the prognosis of BC patients requires further investigation.

Disparities in BC outcomes between Caucasian and African American patients [37–40] as well as the involvement of the CaSR in cancer progression [41, 42] have been amply reported. As expected and reported previously, [17, 32–36], the magnitude of the differences in circulating calcium observed in this study were modest. Our observation that circulating calcium levels in BC cases were higher than those in control subjects is consistent with the potential increase in the synthesis and release of PTHrP by BC cells and the effects of this PTH-like factor on bone resorption [29]. Meanwhile, our finding that circulating calcium levels in African American control subjects are higher than those in Caucasians is intriguing but supports the possibility that the aggressive nature of breast carcinoma in some African American patients may be driven at least in part, by high circulating calcium-dependent mechanisms. Surprisingly, the higher calcium levels in African American patients does not seem to be due to the expression of inactivating CaSR variants at the rs1801726 SNP which is more common in these subjects. One possible explanation for the lack of association between circulating calcium and polymorphisms at the CASR 1801726 SNP may be the generally reported smaller numbers of African American cases in the BioVU and other databases [43]. Overall, this suggests that the high circulating calcium levels in African Americans may be due to other factors that alter systemic calcium homeostasis including the release of calcium stimulated osteolytic factors by normal and/or malignant breast tissues [29], and active vitamin D. Unfortunately, PTH and PTHrP were not part of routine clinical tests and only a subset of patient serum chemistries included active vitamin D analysis from the control and BC case cohorts with genotyping data. Therefore, the confounding effects of PTHrP [10] or Vitamin D [44] as cancer promoting calciotropic hormones could not be evaluated.

It is well established that the CaSR is invariably mutated especially in parathyroid diseases [13, 14]. Our study focused on rs1801725 and rs1801726 which are well characterized inactivating mutations of the receptor in exon 7 [34, 35, 45] to either support their association with CIH or high calcium as an underlying factor for the obvious disparities in the progression of BC in Caucasians and African American patients. Interestingly, other SNPs e.g. rs1751221 [46] and rs112594756 [47] have been shown to correlate with BC susceptibility and prognosis. Although these intronic polymorphisms may affect the expression levels of the receptor, it is unlikely that they are relevant in the overall sensitivity of the mature receptor to calcium and/or the association of the receptor with CIH. Hypercalcemia in patients with advanced and/or metastatic disease has been reported to be strongly associated with poor prognosis [48] while inactivating mutations of the CaSR in exon 7 promoted the development of hypercalcemia in a xenograft mouse model of human squamous cell lung carcinoma [16]. Although the level of serum calcium in low grade BC patients may not be a prognostic indicator for survival, it is possible that the development of hypercalcemia in 10–30% of BC patients without evidence of skeletal metastases [49, 50] may at least in part be due to the expression of inactivating CaSR mutations especially at the rs1801725. Contrary to previous studies showing that both the A986S and Q1011E variants of the CaSR are associated with calcium [34, 35], our findings suggest that polymorphisms at the rs1801725 SNP are more important than those at the rs1801726 SNP in the development of CIH and the associated BC outcomes.

### Limitations of the study and conclusions

The objective of this study was to determine if differences in circulating calcium and the expression of inactivating CaSR mutants in BC patients could shed more light on the causes of the highly aggressive disease in African American patients. Unfortunately, the fewer African American BC cases with both calcium test and GWAS data in the BioVU databases led to inconclusive interpretation of the relationship between circulating calcium and polymorphisms at the rs1801726 SNP. Vitamin D (1,25-dihydroxy vitamin D) levels were not available for most of the cases and control subjects and therefore, could not be considered as a confounding variable. Also, the documented lab calcium tests used in this study were total calcium rather than ionized calcium, the actual ligand for the CaSR. Consequently, it was not possible to relate the potential CaSR activity to the prevailing ionized calcium levels especially in BC patients. Another interesting question which could not be addressed in this study is the effect of these SNPs on calcium levels in BC patients with. This will require a larger, multi-site study to establish not only a better understanding of the role of high circulating ionized calcium but also the impact of inactivating CaSR mutants in BC cases with poor prognosis versus those with favorable prognosis. Overall, this retrospective case-control study reveals that decreased sensitivity of the CaSR to calcium due to inactivating polymorphisms at rs1801725 may predispose BC patients to high circulating calcium-driven larger or aggressive breast tumors.
